# Abnormal blood pressure among individuals evaluated for tuberculosis infection in a U.S. public health tuberculosis clinic

**DOI:** 10.1017/S0950268824001262

**Published:** 2024-10-24

**Authors:** Trevor M. Stantliff, Argita D. Salindri, Rocio Egoavil-Espejo, Ashton D. Hall, Laura Medina-Rodriguez, Kavya Patel, Matthew J. Magee, Elaine M. Urbina, Moises A. Huaman

**Affiliations:** 1 Division of Infectious Diseases, Department of Internal Medicine, University of Cincinnati College of Medicine, Cincinnati, OH, USA; 2 Division of Infectious Diseases and Geographic Medicine, Department of Medicine, Stanford University School of Medicine, Stanford, CA, USA; 3 Department of Epidemiology, Emory University Rollins School of Public Health, Atlanta, GA, USA; 4 Cincinnati Children’s Hospital Medical Center, The Heart Institute, Cincinnati, OH, USA

**Keywords:** hypertension, quantitative interferon-γ, release assays measures, systolic blood pressure, tuberculosis infection

## Abstract

Tuberculosis infection (TBI) has been associated with increased cardiovascular risks. We aimed to characterize abnormal blood pressure (BP) readings in individuals with TBI. We conducted a retrospective study of adults with TBI presenting for their initial medical visit at a large midwestern U.S. public health clinic between 2019 and 2020. Abnormal BP was defined as having a systolic BP ≥ 130 mmHg and/or a diastolic BP ≥ 80 mmHg. Of 310 individuals with TBI, median age was 36 years (interquartile range 27–48), 34% were male, 64% non-US-born; 58 (18.7%) were previously diagnosed with hypertension. The prevalence of any hypertension (i.e., had a history of hypertension and/or an abnormal BP reading) was 64.2% (95% confidence interval 58.7–69.4). Any hypertension was independently associated with older age, male sex, higher body mass index, and individuals of Black race. In conclusion, any hypertension was present in over half of the adults evaluated for TBI in our clinic. Established hypertension risk factors were also common among this group, suggesting that individuals with TBI could benefit from clinical and public health interventions aiming to reduce the risk of future cardiovascular events.

Despite the steady decline in the burden of TB disease in the United States, the prevalence of tuberculosis infection (TBI) has remained largely unchanged at 5% for the past three decades and disproportionately affects immigrant populations [1]. While the risk of progression of TBI into TB disease remains a major concern for TB surveillance and control programs, there is a growing appreciation of the interplay between TBI and non-communicable diseases. For instance, recent studies reported that individuals with TBI have increased rates of obstructive coronary artery disease, myocardial infarction, and diabetes mellitus incidence compared to their non-TBI counterparts [2–4].

TBI has been linked with systemic inflammation and immune activation, which may contribute to increased cardiovascular risk including developing hypertension [5, 6]. Monocytes from individuals with TBI exhibit pro-inflammatory alterations, including enhanced production of interleukin (IL)-6 and tumour necrosis factor (TNF)-α and increased expression of CX3CR1, a chemokine receptor implicated in monocyte migration, endothelial dysfunction, and atherosclerosis formation [5, 7].

A recent analysis by Salindri and colleagues of the National Health and Nutrition Examination Survey cohort (NHANES) reported a higher prevalence of hypertension, a key risk factor for cardiovascular diseases, among civilian, non-institutionalized individuals with TBI in the United States when compared to the general NHANES cohort (58.5% vs. 48.9%) [8]. However, little is known whether hypertension/abnormal blood pressure (BP) (i.e., any hypertension) is more common among those with TBI presenting to public health clinics that manage TBI. In the present study, we aimed to estimate the prevalence of any hypertension and to determine if traditional risk factors for hypertension are similar among persons presenting for TBI care in a public health TB clinic setting. We also explored the relationship between quantitative measures of interferon-γ release assays (IGRA) and systolic and diastolic blood pressure.

We conducted a retrospective cohort study of adults (≥18 years old) who were referred to the Hamilton County Public Health (HCPH) clinic, a large midwestern US public health clinic located in Cincinnati, Ohio, for TBI evaluation between 2019 and 2020. Eligible study participants included those referred to HCPH from community, private, and academic outpatient clinics and were determined to have TBI. Individuals were defined as having TBI based on a positive IGRA or tuberculin skin test (TST), negative chest X-ray findings for TB disease, and a negative clinical evaluation by a medical provider at HCPH. We excluded individuals with history of active tuberculosis or previous TBI treatment. The initial study utilizing this cohort was designed to evaluate the effects of COVID-19 pandemic restrictions in the TBI cascade of care, and those results have been presented elsewhere [8]. For the present analyses, we included sociodemographic and clinical data from all individuals who had available information on hypertension status and BP readings at their initial TBI visit at HCPH.

We defined the history of hypertension by patient’s self-report of prior diagnosis by healthcare providers and documented during the HCPH clinical encounter. BP at HCPH was measured while the patient was sitting and still, according to primary care guidelines. BP was recorded as systolic and diastolic measurements in mmHg. Abnormal BP was defined by having a single reading of systolic BP ≥ 130 mmHg, or a diastolic BP ≥ 80 mmHg. Our primary study outcomes, any hypertension, was defined as having either a history of hypertension or abnormal BP levels at the initial visit.

Self-reported demographics, social risk factors, and clinical characteristics including tobacco use, intravenous (IV) drug use, history of homelessness and incarceration, current medications, as well as BCG vaccination status were determined by chart reviews. Body mass index (BMI) was calculated and classified with BMI <18.5 kg/m^2^ considered ‘underweight’, BMI 18.5–24.9 kg/m^2^ considered ‘normal’ and BMI ≥25 kg/m^2^ considered ‘overweight/obese’.

We entered and managed data collected for this project in an online REDCap database hosted at the University of Cincinnati. We used Wilcoxon rank-sum tests to compare continuous variables between hypertension and non-hypertension groups. Similarly, we used Chi-square or Fisher’s exact tests to compare the distribution of categorial variables. We performed robust (or modified) Poisson regression models [9] to estimate the prevalence ratios (PRs) comparing across different individuals’ characteristics and the 95% confidence interval (CI). Regression models with backward elimination technique were used to determine factors associated with any hypertension. Briefly, we began with a full model where all factors were included. Least contributive factors were then removed iteratively until all factors remaining in the model were statistically significant. We also performed a subset analysis among individuals with quantitative IGRA measures to determine whether IGRA quantitative measures are associated with systolic and diastolic BP levels. We performed linear regression models to explore the relationship between (1) nil count, (2) TB antigen 1-nil, (3) TB antigen 2-nil, and (4) mitogen-nil values and systolic and diastolic BP. We reported beta estimates and corresponding 95% confidence intervals (95%CI) as well as *R*^2^ values to describe how well quantitative IGRA measures describe the observed systolic and diastolic BP data. All analyses were performed using Stata software (v12.0; StataCorp, College Station, TX) and R Statistical Software (v4.3.1; R Core Team 2023) with two-sided *p*-value <0.05 considered significant in all analyses.

Of the 312 individuals with TBI presenting to the public health TB clinic for an initial visit with the medical provider, 310 (99.4%) had a hypertension status and recorded systolic and diastolic BP in their chart. Of 310 individuals with available BP readings, median age was 36 years (Interquartile range [IQR], 27–48), 34% were male, 64% were non-US-born. Of these, 192 (61.9%) patients had an elevated BP (systolic BP ≥ 130 mmHg or diastolic BP ≥ 80 mmHg) (Supplementary Figure S1). Of the 192 patients with an elevated BP reading, only 51 (26.6%) had an established diagnosis of hypertension per history, while 141 others (73.4%) were newly diagnosed with elevated systolic or diastolic BP. There were seven patients with known history of hypertension who did not present with elevated BP at their initial visit. Thus, a total of 199 of 310 patients (64.2%, 95%CI 58.7–69.4) had either a known diagnosis of hypertension and/or an abnormal BP reading during their initial visit and were defined as the group of interest for downstream analyses.

Table 1 shows the characteristics of the study population stratified by BP status. The median age among those with TBI and any hypertension (median = 41, IQR 30–52) were significantly higher compared to those without hypertension (median = 29, IQR 25–39, median difference = 12 years, *p*-value <0.001). In the unadjusted model, the prevalence of hypertension was significantly higher among the older age group (crude prevalence ratio [cPR] = 1.46, 95%CI 1.22–1.74 for those aged 40–59 years and cPR = 1.78, 95%CI 1.50–2.12 for those aged ≥60 years), male (cPR 1.31, 95%CI 1.12–1.54), individuals of Black race (cPR = 1.53, 95%CI 1.28–1.83), with BMI ≥25kg/m^2^ (cPR = 1.47, 95%CI 1.17–1.85), current/former smokers (cPR = 1.36, 95%CI 1.16–1.59), and those with diabetes mellitus (cPR = 1.38, 95%CI 1.14–1.67) (Table 1). The prevalence of hypertension among foreign born was 0.65 times the prevalence among US-born (95%CI 0.56–0.76). In the final multivariable model, the adjusted prevalence of hypertension remained significantly higher among those aged ≥60 years (adjusted prevalence ratio [aPR] = 1.63, 95%CI 1.03–2.48), individuals of Black race (aPR = 1.48, 95%CI 1.11–1.97), and those with BMI ≥25kg/m^2^ (aPR = 1.47, 95%CI 1.04–2.10).

**Table 1.** Characteristics of individuals evaluated for latent tuberculosis at a large midwestern U.S. public health clinic in 2019–2020 according to blood pressure status at initial visit (*n* = 310)

Participants characteristics	Total population (*n* = 310)	No HTN/Abnormal BP at initial visit *n* (%) = 111 (35.8)	HTN/Abnormal BP at initial visit *n* (%) = 199 (64.2)	Measure of Association	
				Crude PR (95% CI)	Adjusted PR^a^ (95% CI)
Age group					
16–39	176 (56.8)	84 (47.7)	92 (52.3)	Reference	Reference
40–59	105 (33.9)	25 (23.8)	80 (76.2)	**1.46 (1.22–1.74)**	1.31 (0.97–1.78)
60+	29 (9.4)	2 (6.9)	27 (93.1)	**1.78 (1.50–2.12)**	**1.63 (1.03–2.48)**
Sex					
Female	205 (66.1)	86 (42.0)	119 (58.0)	Reference	Reference
Male	105 (33.9)	25 (23.8)	80 (76.2)	**1.31 (1.12–1.54)**	1.28 (0.95–1.71)
Country of birth					
US–born	110 (35.9)	19 (17.3)	91 (82.7)	Reference	
Foreign–born	196 (64.1)	90 (45.9)	106 (54.1)	**0.65 (0.56–0.76)**	
*Missing*	4	2	2		
Race					
Black	151 (49.3)	74 (49.0)	77 (51.0)	**1.53 (1.28–1.83)**	**1.48 (1.11–1.97)**
Other race	155 (50.7)	34 (21.9)	121 (78.1)	Reference	Reference
*Missing*	4	3	1		
Tobacco use					
Never smoker	236 (78.7)	95 (40.3)	141 (59.7)	Reference	
Current/Former smoker	64 (21.3)	12 (18.8)	52 (81.2)	**1.36 (1.16–1.59)**	
*Missing*	10	4	6		
Diabetes mellitus					
No	288 (92.9)	108 (37.5)	180 (62.5)	Reference	
Yes	22 (7.1)	3 (13.6)	19 (86.4)	**1.38 (1.14–1.67)**	
Body mass index (*n* = 296)					
Underweight/Normal	89 (30.1)	46 (51.7)	43 (22.6)	Reference	Reference
Overweight/Obese	207 (69.9)	60 (29.0)	147 (71.0)	**1.47 (1.17–1.85)**	**1.47 (1.04–2.10)**
*Missing*	14	5	9		
Prior BCG vaccination					
No	129 (41.6)	33 (25.6)	96 (74.4)	Reference	
Yes	98 (31.6)	41 (41.8)	57 (58.2)	**0.78 (0.64–0.95)**	
Unknown	83 (26.8)	37 (44.6)	46 (55.4)	**0.75 (0.60–0.93)**	

BCG, Bacillus Calmette-Guérin; BP, blood pressure; HTN, hypertension; PR, prevalence ratio; US, United States. Bold indicates that the finding is statistically significant with *α* = 0.05.

a Variables in the full model included age group, sex, country of birth, being Black race, tobacco smoking, diabetes mellitus status, BMI category, prior BCG vaccination, history of intravenous drug use, hyperlipidemia, currently on medication for other diseases, and history of imprisonment; but results were only shown for variables included in the final model selected with the stepwise backward selection.

Among participants with IGRA results available, 72% (72/100) had the quantitative IGRA measures recorded. Among this subset, we did not observe a significant correlation between quantitative IGRA measures and systolic BP (Supplementary Figure S2, p > 0.05). For example, for every unit increase in IGRA nil values, the systolic BP increased on average by 0.632 point (95%CI – 4.326–5.591) (Supplementary Figure S2A). Furthermore, for every unit increase in TB antigen 1 – nil values, the systolic BP decreased on average by 0.280 point (95%CI – 2.289–1.730) (Supplementary Figure S2B). Similarly, for every unit increase in TB antigen 2 – nil and mitogen – nil values, the systolic BP decreased on average by 0.971 (95%CI – 2.709–0.767) and 0.147 (95%CI – 0.577–0.282), respectively (Supplementary Figure S2C,D). We also did not observe a significant correlation between quantitative IGRA measures and diastolic BP (Supplementary Figure S3, p > 0.05). For example, for every unit increase in IGRA nil values, the diastolic BP increased on average by 1.34 point (95%CI – 2.404–5.083) (Supplementary Figure S3A). Furthermore, for every unit increase in TB antigen 1 – nil values, the diastolic BP decreased on average by 0.389 point (95%CI – 1.908–1.129) (Supplementary Figure S3B). Similarly, for every unit increase in TB antigen 2 – nil and mitogen – nil values, the diastolic BP decreased on average by 0.569 (95%CI – 2.005–0.868) and 0.0702 (95%CI – 0.389–0.249), respectively (Supplementary Figure S3C,D).

Our study found that nearly two-thirds of adults with TBI presenting to a public health TB clinic setting in a low-TB endemic area had an abnormal BP reading and/or history of hypertension. These findings are consistent with a recent NHANES report [10], although our estimated prevalence was slightly higher relative to NHANES estimates. This suggests that the magnitude of association between TBI and hypertension may be different across different sub-group populations, especially those presenting in the clinical settings. While this data further supports the previously reported association between TBI and hypertension [8], the relationship between quantitative IGRA values and hypertension has not yet been reported. We did not observe a significant linear association between quantitative IGRA values and prevalence of hypertension.

Our study findings highlight the need to integrate existing non-communicable disease screening and prevention services (e.g., promotion of healthy lifestyle and smoking cessation/counselling programs) with TB control programs. Notably, many patients presented to the clinic for required TB testing (immigration process, healthcare worker screening, etc.) and did not have reliable primary care. Furthermore, patients with known hypertension that presented to our clinic with elevated BP, may require optimizing their antihypertensive management. Since the duration of preferred TBI treatment regimens is 3 to 4 months long, TB programs may offer a unique opportunity to aid in the diagnosis and treatment of hypertension and other chronic diseases with subsequent linkage to primary care services for further management.

We also reported common risk factors of any hypertension as reported in NHANES which includes older age, obesity, and people of Black race. As these key factors were also reported as risk factors of TBI in the US population [1], it is critical to follow-up these individuals or establish linkages to cardiovascular disease/other chronic non-communicable disease clinics to prevent further progression or more severe form of any cardiovascular events. Future studies need to consider designs and methods to isolate the effect of shared common risk factors for TBI and hypertension that may partially explain the observed association between TBI and hypertension in the present study. A prior retrospective cohort study showed a two-fold increased risk of incident hypertension among individuals with TBI compared to non-TBI controls. The risk of incident hypertension was attenuated after TBI treatment [6].

Our study is subjected to several limitations. First, we could not confirm a hypertension diagnosis in our cohort. Our study used single-visit BP measurements, which are prone to measurement/information bias. Published studies suggested that blood pressure levels may be elevated during a clinic visit due to anxiety (i.e., white coat syndrome), and affected 25–30% of patients in outpatient clinics [11]. Further, self-reported previous hypertension diagnosis is also prone to recall bias. This warrants further clinical investigation with larger prospective studies to better characterize BP readings, including comparison between those who were treated for TBI versus those who were not. Second, since we relied on medical charts, we did not have access to other correlates of hypertension and TBI risk that may affect the association between TBI and hypertension such as stress, family history, diet, and lifestyle. Consequently, our estimates may be slightly distorted from unmeasured confounding effects. Third, we conducted our study in a single public health clinic in Ohio, preventing us to draw any inference to other clinic settings, especially with different population characteristics that may affect the background prevalence of TBI and hypertension in the population level.

Despite study limitations, our study provides epidemiologic evidence suggesting that hypertension is common among individuals with TBI. Integrating TB control program with non-communicable care could improve quality and individuals’ access to care to lower the burden of the two diseases through more centralized public health interventions.

## Supporting information

Stantliff et al. supplementary material Stantliff et al. supplementary material

## Data Availability

Data that supports the findings of this study can be made available upon request pending review by the local public health clinic.

## Supplementary material

For supplementary material accompanying this paper visit http://doi.org/10.1017/S0950268824001262. click here to view supplementary material
