# Supplementary material for: Abnormal blood pressure among individuals evaluated for tuberculosis infection in a U.S. public health tuberculosis clinic
Source: Epidemiol Infect. 2024 Oct 24;152:e133. doi: 10.1017/S0950268824001262 (PMC11502439; doi:10.1017/S0950268824001262)

**Epidemiology and Infection**

**Title:**

Abnormal Blood Pressure Among Individuals Evaluated for Tuberculosis Infection in a U.S. Public Health Tuberculosis Clinic

**Authors:**Trevor M. Stantliff*,^1^ Argita D. Salindri*,^2^ Rocio Egoavil-Espejo,^1^ Ashton D. Hall,^1^ Laura Medina-Rodriguez,^1^ Kavya Patel,^1^ Matthew J. Magee,^3^ Elaine M. Urbina,^4^ Moises A. Huaman^1^

**Supplementary Material**

**Supplementary Figure S1. Diagram flow to depict the study inclusion of individuals presenting for TBI at a large midwestern US public health clinic, N=310**


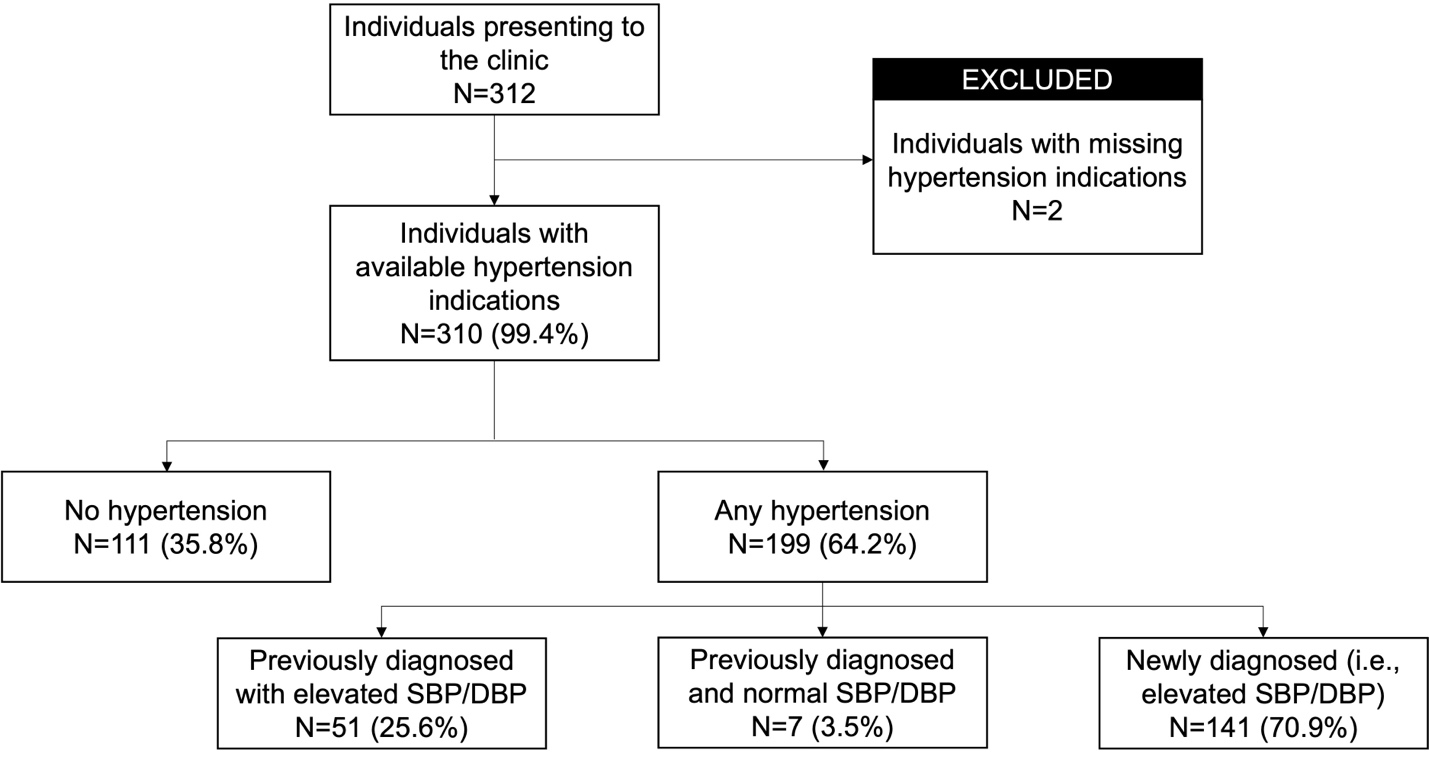


**Supplementary Figure S2. Results from linear regression models to estimate the relationship between systolic blood pressure and A) nil count, B) TB antigen 1 – nil, C) TB antigen 2 – nil, and D) mitogen - nil**


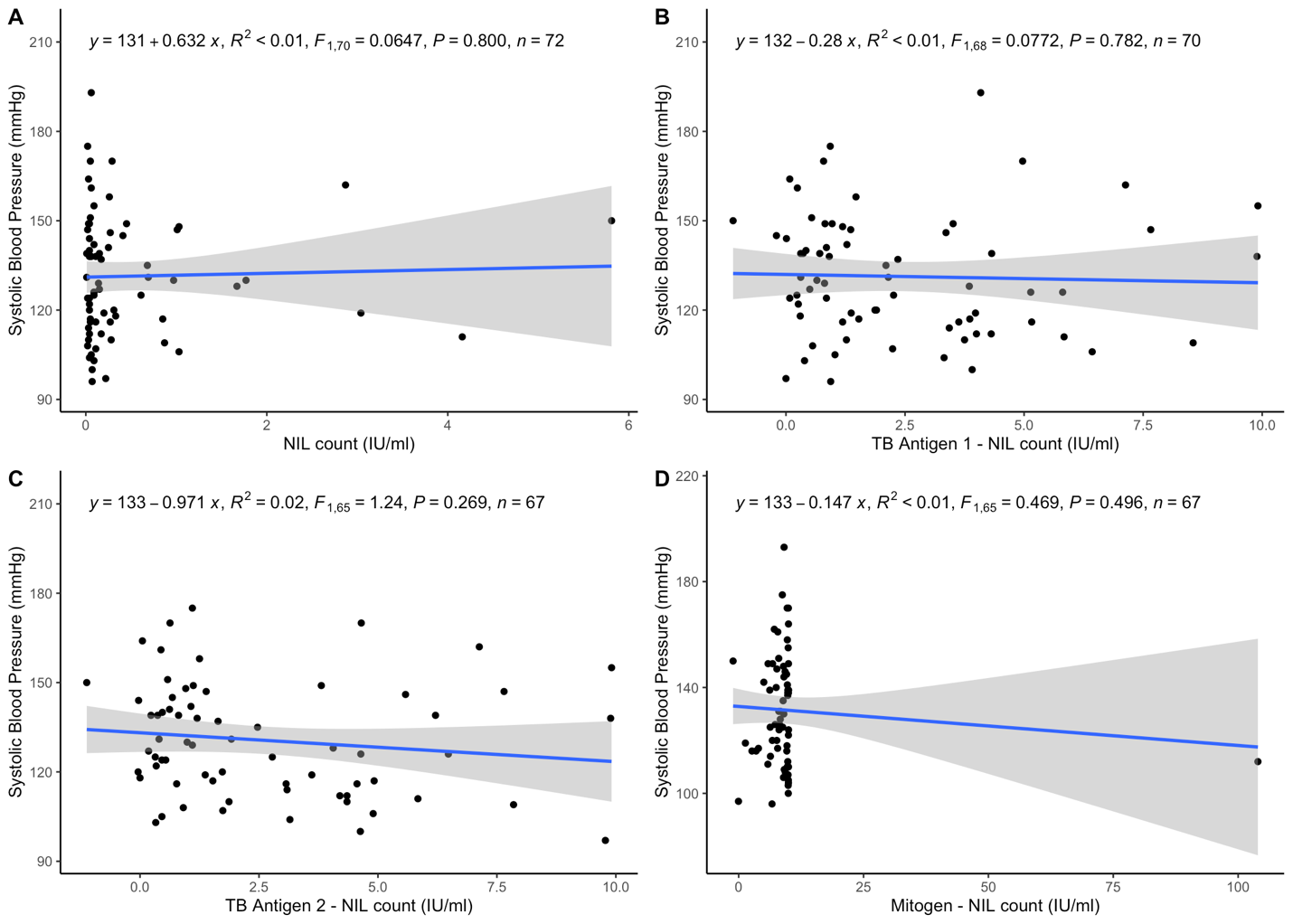


**Supplementary Figure S3. Results from linear regression models to estimate the relationship between diastolic blood pressure and A) nil count, B) TB antigen 1 – nil, C) TB antigen 2 – nil, and D) mitogen – nil**


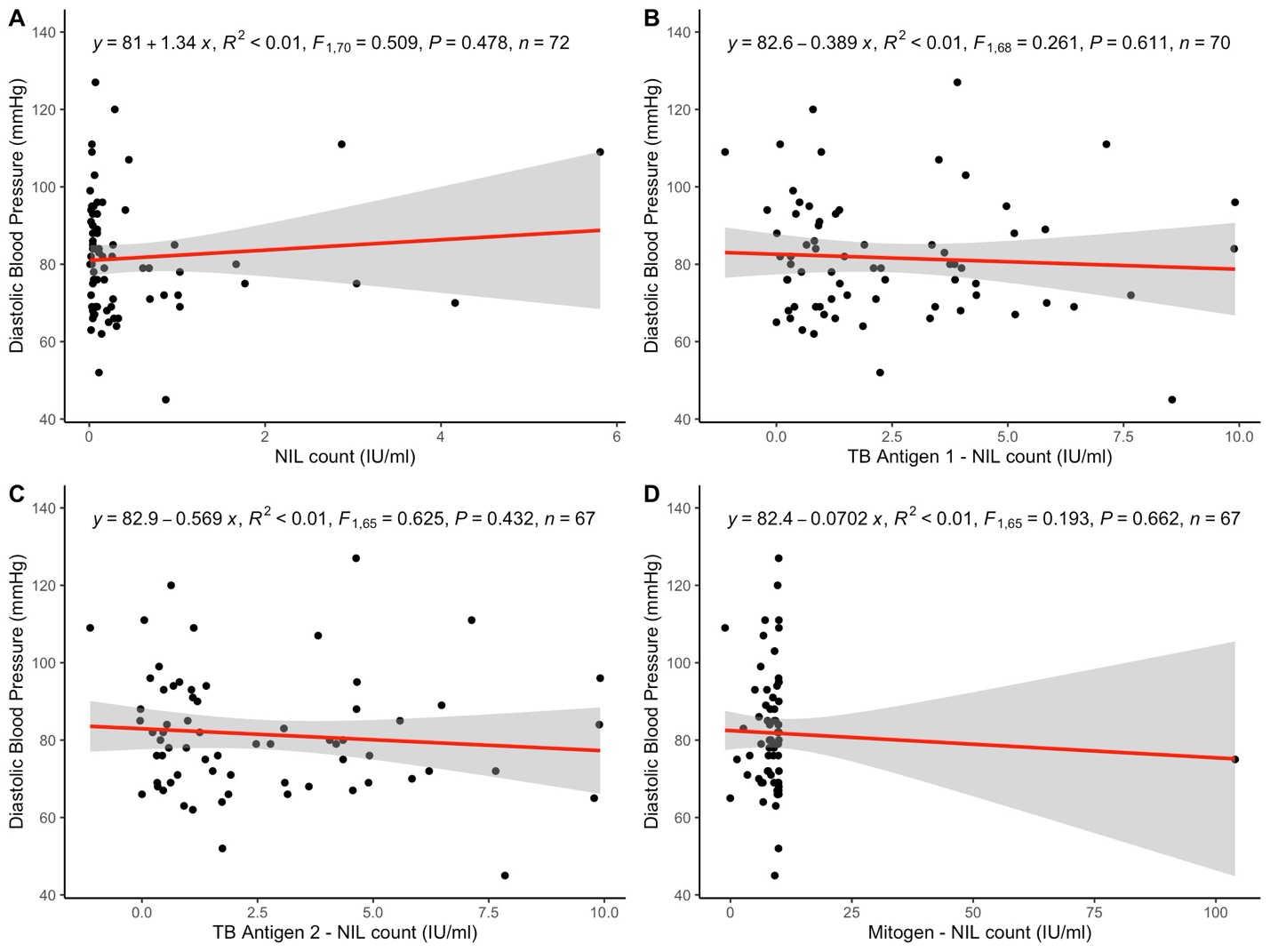

Supplement: Stantliff et al. supplementary material [file S0950268824001262sup001.docx]
